# Emotions Associated With Urinary Incontinence in Nursing Home Residents: A Phenomenological Analysis of Healthcare Professionals' Perspectives

**DOI:** 10.1002/nop2.70620

**Published:** 2026-06-22

**Authors:** Meltem Yildirim, Sandra Rierola‐Fochs, Eduard Minobes‐Molina, Laura Coll‐Planas, Pau Moreno‐Martin, Suzanne Hagen, Vinicius Rosa Oliveira, Javier Jerez‐Roig

**Affiliations:** ^1^ Research Group on Methodology, Methods, Models and Outcomes of Health and Social Sciences (M_3_O), Faculty of Health Sciences and Welfare, Centre for Health and Social Care Research (CESS), University of Vic‐Central University of Catalonia (UVic‐UCC) Vic Spain; ^2^ Institute for Research and Innovation in Life Sciences and Health in Central Catalonia (IRIS‐CC) Vic Spain; ^3^ Research Centre for Health, School of Health and Life Sciences, Glasgow Caledonian University Glasgow UK; ^4^ Institute of Sport Science and Innovations, Lithuanian Sports University Kaunas Lithuania

**Keywords:** emotions, healthcare provider, nursing homes, qualitative research, urinary incontinence

## Abstract

**Aim:**

To describe the emotions of nursing home (NH) residents associated with urinary incontinence (UI) from the point of view of healthcare professionals (HCPs).

**Design:**

A phenomenological descriptive study.

**Methods:**

Seventeen HCPs caring for older people with UI for at least 6 months participated across 9 NHs in the Osona county (Barcelona, Spain). Individual interviews were conducted using online videoconferencing and, with participant consent, each session was recorded. Data obtained were analysed with Colaizzi's 7‐stage method, using descriptive phenomenology. In‐depth thematic analysis was finalised by using ATLAS.ti 8.0.

**Results:**

UI in NH residents triggers negative emotions including feelings of impotence, guilt, frustration, embarrassment, anger, discomfort, worry and insecurity. Alleviating factors include de‐emphasising UI, implementing adaptations in NHs, and building a strong bond of trust between NH residents and HCPs. Conversely, resident conflicts and cognitive impairment worsen these negative emotions.

**Conclusion:**

HCPs perceive a wide range of negative emotions related to UI among NH residents, influenced by cognitive impairment and interpersonal conflicts. These emotions can be eased through appropriate person‐centred strategies.

**Implications for the Profession and/or Patient Care:**

There is a need to intentionally incorporate person‐centred, emotionally attuned care strategies to reduce residents' distress and improve continence in NH.

**Impact:**

This study examined how UI generates negative emotions among NH residents from the perspective of HCPs. There is a spectrum of distressing emotions related to UI that are either mitigated or exacerbated by contextual and relational factors. These findings can inform caregiving approaches in NH by guiding more tailored and empathetic strategies to support residents living with UI.

**Reporting Method:**

Consolidated criteria for reporting qualitative research.

**Patient or Public Contribution:**

HCPs provided their perspectives on the emotions associated with UI in NH residents.

**Abbreviations**
HCPs: healthcare professionals
NHs: nursing homes
UI: urinary incontinence

## 1 Introduction

Urinary incontinence (UI) is a geriatric syndrome characterised by the involuntary loss of urine, mostly affecting women, with prevalence rates ranging from 25% to 45% across different regions of the globe (Batmani et al. 2021). It is frequently associated with cognitive impairment, physical inactivity, and immobility syndrome, among other factors, and causes an important burden from a societal (high costs) and an individual point of view (Farrés‐Godayol et al. 2022; Milsom and Gyhagen 2019). UI is considered more bothersome in long‐term care than in other settings, hindering quality of life, autonomy, dignity, mood and social interactions (Karakaya et al. 2019; Ostaszkiewicz et al. 2018).

Most older adults and even healthcare professionals (HCPs) mistakenly believe that incontinence is part of the normal ageing process and/or is an irresolvable problem (Bascur‐Castillo et al. 2019; Hunter and Wagg 2018; Yan et al. 2022). These misconceptions, together with gaps in continence‐care knowledge among direct care staff, can negatively influence the quality of care and the implementation of treatment plans, potentially increasing the frequency of UI and affecting residents' quality of life (Hunter and Wagg 2018; Borglin et al. 2020). Therefore, in clinical practice it is essential to prioritise staff education on effective continence care to foster resident‐centred approaches and preserve dignity (Ostaszkiewicz et al. 2018).

## 2 Background

Older people living in nursing homes (NHs) represent a frail segment of the population, with a high prevalence of dementia, and more than 70% presenting with some type of cognitive impairment (Farrés‐Godayol et al. 2022). As a result, most NH residents are unable to self‐report or respond reliably to questionnaires assessing their health status or related feelings (Farrés‐Godayol et al. 2022). They typically rely on staff assistance to access the toilet and achieve successful voiding (Newman 2019).

Beyond the physical discomfort it causes, UI provokes a range of emotional responses among NH residents. Feelings of embarrassment, shame, frustration and anxiety are commonly reported by individuals experiencing UI and can significantly affect overall quality of life, self‐esteem and social interactions (Yan et al. 2022; Javanmardifard et al. 2022). Thus, to provide holistic health care, NH staff must acquire a deep understanding of the emotional experiences associated with UI and the ways these emotions shape residents' well‐being.

## 3 The Study

### 3.1 Aim and Objective

To explore the emotions experienced by NH residents with UI as perceived by HCPs, and to identify the factors that alleviate or worsen these emotional responses.

## 4 Methods

### 4.1 Design

The present study used a descriptive phenomenological design (Sundler et al. 2019) to investigate the NH residents' emotions associated with UI, from the perspective of HCPs responsible for their care. This design, which takes its roots from Husserl's philosophical approach, aims to extract the essence of an experience in order to understand its fundamental structure by putting aside the classical presuppositions of doing philosophy and conducting a detailed description of lived experiences to determine their meanings for themselves (Wirihana et al. 2018). Therefore, descriptive phenomenology based on Husserl's philosophical background was considered the most appropriate methodological approach for allowing us to deeply understand UI‐related subjective emotional experiences, as observed and interpreted by HCPs.

### 4.2 Theoretical Framework

This investigation forms an integral part of the broader OsoNaH Project (Farrés‐Godayol et al. 2021) that employs a mixed‐methodology approach to comprehensively examine diverse aspects related to UI in NHs, which is registered in Clinical Trials with the identification number NCT04297904 (Farrés‐Godayol et al. 2021). This study is grounded in Husserl's descriptive phenomenology, which seeks to describe the essence of lived experience through careful attention to participants' accounts and the reduction of researcher preconceptions. This approach is well aligned with our aim, since the emotions associated with UI in NHs are often communicated through behaviours, interactions and embodied responses rather than direct verbal expression (Murphy et al. 2021). By drawing on the close observational experience of HCPs, the study accesses meaningful insights into how residents' emotional experiences are perceived and interpreted in everyday care.

### 4.3 Sampling and Recruitment

The study took place in 9 NHs in Osona (Barcelona, Spain). The participating facilities included privately owned nursing homes, non‐profit foundations, and mixed‐management centres operating under public contracts with a substantial proportion of publicly funded beds. The homes were generally small to medium‐sized (approximately 25–100 residents) and were distributed across both urban settings and rural municipalities within the Osona region. We initially aimed to explore UI‐related emotions by conducting in‐person interviews with NH residents without cognitive impairment. However, due to the restrictions implemented during the COVID‐19 pandemic, we opted to invite HCPs to share the emotions they observed in the residents (both with and without cognitive impairment).

A purposive sampling strategy with maximum variation was employed to recruit HCPs with diverse backgrounds and experiences related to residents' emotional responses to UI. Variation was sought across nursing home type, gender, age, professional category, and years of experience. This approach ensured the richness and depth of the data and is consistent with a descriptive phenomenological design, which prioritises capturing a wide range of lived experiences rather than achieving statistical representativeness. In total, 25 HCPs were invited to participate via email; of these, six did not respond and two declined participation for personal reasons.

### 4.4 Sample Size and Power

The sample size was guided by the principle of data saturation, whereby data collection continued until no new themes or relevant insights emerged from the individual interviews. Saturation was assessed iteratively throughout the data collection and analysis process. New meaning units and emotional categories continued to emerge up to interview 15, whereas interviews 16 and 17 yielded no additional substantive findings, suggesting that thematic saturation had been achieved. This conclusion was supported by repeated review of the meaning units and systematic comparison of newly generated data with previously established categories.

### 4.5 Population and Sample

The population of interest consisted of HCPs working in NHs in the Osona region (Barcelona, Spain). These professionals provide direct and continuous care to residents living with UI and are therefore well positioned to observe and interpret residents' emotional responses in daily care situations. The final sample included nurses, nurse's aides, physiotherapists, occupational therapists, psychologists and one nursing home director, reflecting the multidisciplinary nature of continence care in these settings. To capture a wide range of perspectives on residents' emotional experiences, we purposefully sought variation in the type of nursing home, participants' gender, age, years of professional experience and professional category. This heterogeneity was intended to capture a broad range of perspectives on how residents express emotions related to urinary incontinence.

### 4.6 Data Abstraction

All interviews were transcribed verbatim by a member of the research team. The first author (M.Y.) reviewed each transcript for accuracy, completeness and anonymisation. After verification, transcripts were organised within ATLAS.ti 8.0 to facilitate systematic data handling. An initial reading of all transcripts was conducted to gain a general understanding of the data. Subsequently, significant statements related to residents' emotional experiences of UI were identified and highlighted. These statements were then grouped into meaning units, which were condensed and abstracted into codes. Codes were compared for similarities and differences and organised into categories and overarching themes that captured the essence of the phenomenon under study.

The analysis was iterative, involving continuous movement between the whole dataset and its parts to ensure that interpretations remained grounded in the data. This process supported an in‐depth exploration of participants' accounts and the development of a rich description of the phenomenon. To enhance rigour, more than one researcher was involved in the analytical process. Coding and emerging themes were discussed within the research team until consensus was reached, ensuring consistency and credibility of the findings.

### 4.7 Inclusion and Exclusion Criteria

HCPs were eligible to participate if they met the following conditions: (i) voluntary participation in the study, and (ii) a minimum of 6 months of experience providing care to residents with UI in a NH. Therefore, temporary or administrative staff without regular contact with residents were excluded.

### 4.8 Data Collection

Subjects voluntarily agreed to participate and signed the informed consent form electronically. Afterwards, they received an email with the semi‐structured interview guide (Table 1) and a guide outlining how the individual interviews would be conducted. These materials were provided to ensure that participants were well‐informed and prepared for their involvement in the study.

**TABLE 1:** Semi‐structured interview guide.

Main questions	Alternative questions^a^
From your point of view, how do residents perceive having urinary incontinence?	– Why do you think they perceive it that way?
	– What do you think makes them perceive that way?
	– Which emotions do you think are at the base of these thoughts?
What kind of emotional support do you believe residents with urinary incontinence need?	– Do you think you can completely satisfy these needs? If not, why?
	– Are there any challenges in providing the emotional support they need?
	– Are there any facilitators in providing the emotional support they need?
Do you observe any conflicts among residents due to one of them having urinary incontinence?	– What do you think is the main cause of this conflict?
	– How do you think this conflict affects the resident's emotional state?
How do you observe the effect of urinary incontinence on residents' daily activities?	– What type of activities are affected?
	– How do you think this change in daily activities emotionally affects residents with urinary incontinence?

^a^ These questions were selectively used when the respondent's initial response did not provide sufficient information to thoroughly explore the investigated phenomenon.

Data were collected between the 20 January and 18 March of 2021 through online interviews at home to overcome the restrictions imposed by the pandemic. The interviews were conducted via Zoom videoconference with an approximate duration of 20 min and recorded with the participant's consent. To ensure consistency, the first author (M.Y.) conducted all the interviews to develop familiarity with the data as she was responsible for the data analysis process. She is a registered nurse trained in qualitative research (including descriptive phenomenology) and had no prior relationship with any participant. When the participant was from another discipline (i.e., physiotherapy), other researchers from other disciplines joined the interview session to achieve a broader perspective on the subject being studied and ensure that important questions specific to the participant's discipline were addressed. One researcher (PMM) transcribed all the interviews verbatim. The transcripts were subsequently shared with the participants for any potential comments, although no response was obtained from them.

### 4.9 Data Analysis

The first author (M.Y.) led the analysis process by using a modified version of Colaizzi's 7‐stage method, which draws upon Husserl's descriptive phenomenological approach that aims to describe the essence of lived experiences from the first‐person perspective (Wirihana et al. 2018). In our study, although it was not possible to obtain these experiences from the residents who live with UI, we intended to listen to them from the HCPs who care for UI on a daily basis as they are the ones who observe and manage residents' emotional responses to this condition. Therefore, Husserl's perspective served as a valuable framework for our analysis. After the initial analysis, preliminary meaning units and codes were presented to the other authors to ensure interpretation and comprehensibility of the results. Any discrepancies in coding or interpretation were addressed through discussion among the research team. Differences were examined in relation to the original transcripts and resolved through consensus during peer‐debriefing meetings, ensuring consistency and shared understanding across the analytic process.

**FIGURE 1:**
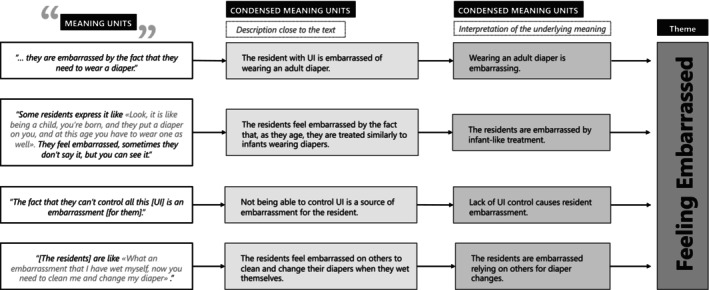
Analysis process including meaning units, condensed meaning units and theme.

Following Colaizzi's analytical steps, significant statements were first identified and then condensed into meaning units that captured their essential content. Meaning units sharing conceptual similarity were grouped into preliminary clusters, which were further refined into subthemes. These subthemes were subsequently synthesised and abstracted into the final overarching themes presented in the Results section. This progression from meaning units to themes is illustrated in Figure 1 and Table S1. Subsequently, an in‐depth content analysis was conducted using ATLAS.ti 8.0. Succeeding the development of the final thematic structure, the themes were returned to participants for member checking. Participants who responded confirmed that the themes reflected their observations of residents' emotional expressions and no changes were requested. This procedure complemented the peer‐debriefing and consensus discussions already undertaken within the research team and contributed to the credibility of the findings.

### 4.10 Ethical Considerations

The study received approval from the Ethics and Research Committee of the University of Vic—Central University of Catalonia (UVic‐UCC) (92/2019 and 109/2020), as well as the Clinical Research Ethics Committee of the Osona Foundation for Health Research and Education (FORES) (2020118/PR249). Participants received written information outlining their right to withdraw at any time without consequences. Electronic informed consent was obtained before scheduling interviews. All data were anonymised using numerical codes, stored in a secure password‐protected server, and accessible only to authorised team members. The coding key linking participants' identities to these numerical codes and the informed consent forms were stored separately from the anonymised dataset to ensure confidentiality. Audio files were deleted after transcription.

### 4.11 Rigour

Data were gathered at a time suitable for the participants through individual interviews in a private environment to ensure confidentiality. The study was carried out across multiple NHs, involving participants from diverse backgrounds, enhancing the applicability and transferability of findings. To ensure data stability, interviews were conducted with HCPs with a minimum of 6 months' experience in caring for individuals with UI, thus allowing for a comprehensive exploration of their first hand experiences of the subject. Peer discussions were held among the authors to further validate the initial findings and strengthen confirmability. A uniform semi‐structured interview guide was used to ensure consistency across interviews. All interviews were conducted by a single trained researcher (M.Y.) to reduce variability in data collection. In addition, an audit trail was maintained to document key methodological and analytical decisions throughout the research process. Adherence to the Standards for Reporting Qualitative Research was ensured throughout the study (Dossett et al. 2021).

The assumptions bracketed by the research team included the beliefs that UI is a common condition that burdens daily care in NHs and residents predominantly experience negative emotions. These assumptions were recorded in the reflexive journal and consciously set aside during analysis.

To enhance reflexivity and ensure alignment with Husserlian descriptive phenomenology, the first author (M.Y.) maintained a reflexive journal throughout the entire research process, documenting pre‐existing assumptions, analytic decisions, and reflections on interaction with participants. Reflexive entries were reviewed and revisited to monitor possible influence on interpretation, supporting the process of bracketing (epoché). Regular peer‐debriefing sessions with co‐authors reinforced reflexive awareness and helped ensure that the analysis remained grounded in participants' accounts rather than researchers' expectations. Although the risk of bias is a common concern in qualitative research due to its subjective nature, the authors made significant efforts to tackle its impact. Participants were selected from diverse genders and professions with varying levels of experience at different NH settings. They were assured of confidentiality and encouraged to share honest and candid observations of residents' emotional responses. To mitigate researcher bias, the first author (M.Y.), who also supervised the analysis process, conducted all interviews. Therefore, consistency was ensured by using the same trained interviewer for all interviews and a uniform semi‐structured guide. Continuous reflexive journaling supported the bracketing of preconceptions.

Additionally, there was a risk of recall bias, an important consideration as HCPs were recalling observations of residents' emotional responses. However, we believe this risk was controlled as participants cared for residents with UI on a daily basis and, therefore, could share both recent and past experiences. Furthermore, the risk of confirmation bias was addressed by involving multiple researchers in the interpretation after the preliminary analysis was completed. Peer‐debriefing sessions and consensus meetings reduced the influence of individual perspectives during coding and theme development. An audit trail was kept to document methodological decisions and enhance confirmability.

## 5 Findings

### 5.1 Participant Characteristics

We included a total of 17 participants, representing the following professions (Table 2): physiotherapists (*n* = 4), psychologists (*n* = 2), nurse's aides (*n* = 5), nurses (*n* = 3), occupational therapists (*n* = 2), and a director (*n* = 1). The mean age of the participants was 39.2 years (SD = 10.8), ranging from 26 to 60 years old. Female (*n* = 14) outnumbered male (*n* = 3), and the participants' academic backgrounds predominantly consisted of bachelor's (*n* = 6) and master's degrees (*n* = 9). The participants worked an average of 14.8 (SD = 10.3) years in their profession and an average experience of 14.3 (SD = 10.4) years caring for residents with UI.

**TABLE 2:** Descriptive characteristics of the participants.

Participant #, Profession	Age (years)	Gender	Last obtained academic degree	Years of experience	
				In the profession	Caring for residents with UI
01, Physiotherapist	32	Male	Bachelor's degree	10	8.5
02, Psychologist	35	Female	Master's degree	7	7
03, Physiotherapist	39	Female	Postgraduate degree	7	7
04, Nurse's aide	40	Female	Vocational education	15	15
05, Nurse's aide	57	Female	Bachelor's degree	7.5	7.5
06, Nurse's aide	27	Female	Vocational education	9	9
07, Psychologist	43	Female	Bachelor's degree	18	18
08, Physiotherapist	26	Male	Bachelor's degree	1	1
09, Occupational Therapist	41	Female	Master's degree	18	18
10, Nurse's aide	52	Female	Vocational education	10	10
11, Physiotherapist	29	Female	Master's degree	6	4
12, Occupational Therapist	51	Female	Master's degree	22	22
13, Director (Nurse)	60	Female	Master's degree	46	46
14, Nurse's aide	51	Female	Vocational education	22	17
15, Nurse	43	Female	Master's degree	23	23
16, Nurse	32	Female	Master's degree	11	11
17, Nurse	43	Male	Bachelor's degree	20	20

### 5.2 Themes

**FIGURE 2:**
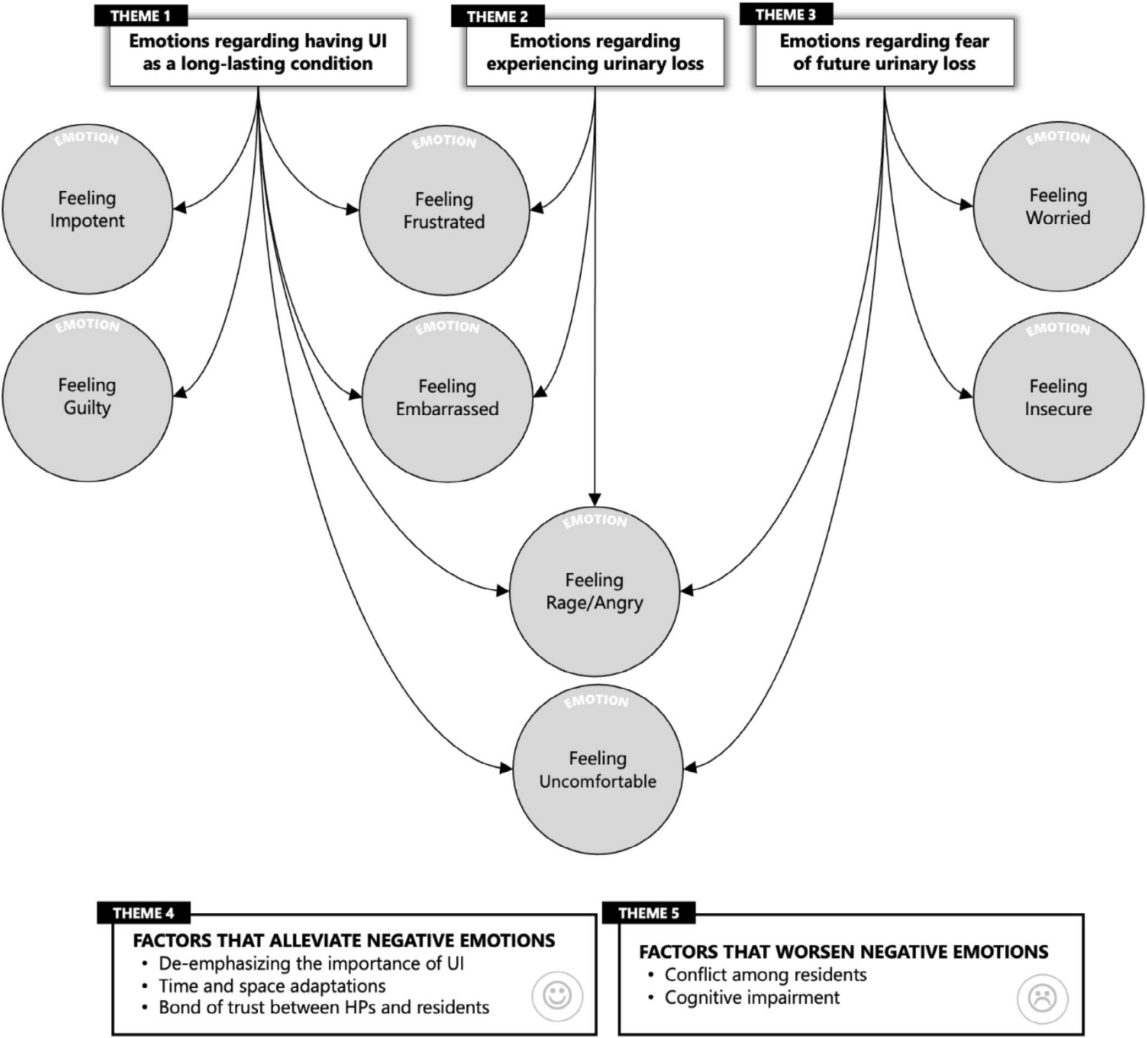
The categories that composed the main themes.

The general results are depicted in Figure 2. From the point of view of the HCPs, the residents experienced various feelings regarding 3 different but related situations: (i) having UI as a long‐lasting condition, (ii) experiencing urinary loss, and (iii) fear regarding future urinary loss. Additionally, there were certain factors that alleviated the negative emotions, such as de‐emphasising UI, implementing adaptations in NHs, and building a strong bond of trust between NH residents and HCPs. Conversely, it was found that conflicts between residents and cognitive impairment were factors that worsened the overall psychological distress of the negative emotions.

#### 5.2.1 Theme 1: Emotions Regarding Having UI as a Long‐Lasting Condition

The HCPs' reflections emphasised that the long‐term nature of UI is not a factor that makes the condition easier to accept with time. Instead, it remains a constant source of embarrassment, frustration, guilt, impotence, discomfort and anger. The HCPs who care for residents with long‐term UI explained their observations regarding its emotional aspect as follows:

Some [residents] are bothered by having UI saying that *«This is a bummer that this has to happen to me»* because they are ***embarrassed*** by the fact that they need to wear a diaper. Some residents express it like *«Look, it is like being a child, you're born, and they put a diaper on you, and at this age you have to wear one as well»*. They feel ***embarrassed***, sometimes they don't say it, but you can see it [in their attitudes]. *—#06, Nurse's aide*

Hmm… [thinking] They [the resident] relate it [UI] to aging or something else? I think [yes] they relate it to being wet and dirty. Something that you had control during all your life, and then [now], like ***frustration***, and a bit of ***rage***, no? They say *«I am dirty, I couldn't hold my pee, I am wet, I am sorry, but you need to clean me because I can't»*, well, this is how they say/show it to us. *—#05, Nurse's aide*

They take it [UI] very badly because of many reasons. Physically they notice that they have a diaper, and when they put on diapers they say *«it makes my ass that big that you notice the lump»*. Also, the diaper gives an ***uncomfortable*** sensation of heat. Then the smell and the sensation of constant humidity; they [the residents] take it all very bad as well. *—#11, Physiotherapist*

They feel very ***impotent***, and very ***frustrated***. The fact that they can't control all this [UI] is an ***embarrassment*** [for them]. *—#02, Psychologist*

The experience of individuals [residents] who were cognitively fine is very bad. It [UI] is like losing some part of your autonomy, your values… It is about accepting that you have a dependency, you need help from others. Nobody of any age would like this, they are losing the capacity to control their life, their environment. Therefore, it emotionally affects them a lot… Especially among the ones [residents] that are cognitively okay, the sensation of ***impotence*** affects them seriously. *—#15, Nurse*

What I see common among residents with UI is that they speak about UI with very few people. They decrease their tone while speaking as if it is [UI] something that we need to speak secretly, that most sure there is no solution and that the only **guilty** is the person who experiences it. They are like *«shut the door, that nobody sees me*… *I am sorry that you need to change my clothes/diaper»*. They say it very frequently, it's more sounds like ***guilt***. *—#15, Nurse*

#### 5.2.2 Theme 2: Emotions Regarding Experiencing Urinary Loss

The HCPs observed that when the residents experience episodes of urinary loss, it triggers feelings of embarrassment, frustration, and anger because they feel that they have failed to be continent in front of the people they know, which results in their need to be changed and cleaned by a HCP.

[The residents] are like *«What an **embarrassment** that I have wet myself, now you need to clean me and change my diaper» —#05, Nurse's aide*

There is always an urgency to arrive at the toilet, and always the fear of wetting themselves, then [if it happens] they feel ***embarrassed*** in front of people they know. *—#11, Physiotherapist*

I think they are really bothered [by having UI]. It's like, yes, they seem that they accept it but, they are not like saying *«Okay!»*, you know? There are people [residents] that accept it in general, but anyways there are moments of ***frustration*** when they realise that they couldn't hold their pee. *—#05, Nurse's aide*

If they have a urine leakage before arriving at the toilet, they get ***angry*** [with themselves]. *—#12, Occupational Therapist*

It affects them [residents] negatively because it is an uncomfortable moment for them. If they notice it in time and can leave, then it's okay, but if they wet themselves, others notice it, and they realize it too. They may become upset with themselves, sometimes even ***angry***, or ***feel bad*** about it. These situations can be quite ***embarrassing***, especially when it happens during an activity.” —#08, Physiotherapist

#### 5.2.3 Theme 3: Emotions Regarding Fear of Future Urinary Loss

The HCPs commented that the residents suffer from a constant fear regarding experiencing urinary loss while participating in certain indoor or outdoor activities. This fear is linked with a range of emotions such as feeling uncomfortable, insecure, and worrying as they contemplate the possibility of a urinary loss episode occurring. They also show anger because frequent toilet visits to prevent possible urinary loss interrupt significant moments such as family visits and social activities.

It [UI] affects their daily activities. It's possible to find a resident who stops doing his/her daily activities because at any moment he/she can leave [the activity] running [to go to the toilet], they ***don't feel comfortable***. *—#01, Physiotherapist*

I know they feel ***uncomfortable***. [Uncomfortable of] participating in an activity or something else. [They are like] *«what if I can't hold my pee and I need to leave?». —#04, Nurse's aide*

When they go out for a walk, of course it is a problem because later they arrive with their pants wet, and it's very distressing for them. When they do a group activity, then of course sometimes they feel a little bit ***insecure*** because they must go running [to the toilet] so the others [the other residents] don't see that he/she has lost urine. *—#17, Nurse*

They [the residents] are ***worried*** if they need to do a certain activity or they need to go to a certain place, they are like *«let's see if I can hold my pee, let's see if I will get myself wet or not…»*. *—#7, Psychologist*

I also remember one resident, who is no longer with us, who was very worried about wetting herself. When she was not attended to in time, she would become very **worried** and start crying, saying: *«Oh, I'm going to wet myself, I'm going to wet myself… I don't want to wet myself.» —#17, Nurse*

They [the residents] are doing an activity for example, and suddenly they have the urgent need to go to the toilet. Or for example, they are having a weekly family visit and right at that moment they need to go to the toilet, this urgency that appears at important moments, they take it with a lot of ***anger***. *—#02, Psychologist*

#### 5.2.4 Theme 4: Factors That Alleviate Negative Emotions Regarding UI

The HCPs underlined that to alleviate negative emotions regarding UI they choose to de‐emphasise the importance of UI in the eyes of the residents. In other words, they verbalise it as a normal condition that comes with ageing and they put into practice certain little adaptations in activity duration and places to help avoid UI episodes. On the other hand, the bond of trust developed between the HCP and the resident is a facilitating factor when the resident needs to explain his/her experience/complaints regarding UI.

***De‐emphasising the importance of UI*** would help them [the residents] emotionally. Giving the message that it's not a problem for us [HCPs], so it shouldn't be a problem for them either. Not adding more problems to something that is already problematic, you know? Speaking to them calmly and friendly like it [IU] is not that important, it's something related to aging, we all will have it at the end. We are putting ourselves in the same boat to make them feel understood and empathised. *—#05, Nurse's aide*

Well, first of all… when you identify it [urine leakage], ***not giving it too much importance*** so that the person feels calm and secure and sees that nothing is happening. *—#07, Psychologist*

***Little adaptations*** like planning the group activities in spaces that are closer to the toilet, shortening the time of activities from 1.5 hours to 45 minutes help them [the residents] ***to feel calm and secure***. *—#02, Psychologist*

I think the biggest facilitator is the ***bond of trust*** we build with them [the residents] day by day. Sharing intimate problems [to a person that you trust] is easier than explaining it to a person you have met recently. When there are new professionals working in the NH, they [the residents] always come to the ones that they have confidence with to explain their problems. I believe the bond of trust they have with us is a great facilitator for an aspect that is precisely so intimate, you know? So, I think that's an advantage. *—#07, Psychologist*

***[Having trust]*** with the person [HCP] who helps you get up, who is with you during the day, is a very important factor; communication also helps. Sometimes, I notice residents who haven't said anything [about their UI] to anybody, and they wait for me for days, and when we do a one‐to‐one activity, they bring up the topic. *—#11, Physiotherapist*

#### 5.2.5 Theme 5: Factors That Worsen Negative Emotions Regarding UI

The healthcare professionals (HCPs) expressed their concern regarding two interrelated factors that exacerbate the negative emotions experienced by the residents because of urinary loss. One of them was the presence of conflict among residents, verbal criticism of individuals experiencing urinary loss by the other residents who witness the situation. Additionally, if the resident who is criticised by the others has cognitive impairment, his/her difficulty in understanding the situation and expressing himself/herself makes it emotionally more challenging for the person.

In the common areas if one [resident] wants to go to the common toilet and it's occupied, he/she starts speaking out loud like *«He/she is always in the toilet, whenever I want to go, never I can»*. There is this kind of ***conflict [among residents]***. … [After a urine loss] we ventilate the room a bit, but for example, one resident starts saying *«Uf! Here it smells very bad!»* and the one with UI feels embarrassed. Even if he/she doesn't tell it clearly, you can see its corporal expression, it's like more closed, more ashamed, like saying *«get me out of here, please!». —#06, Nurse's aide*

There are lots of ***conflicts [among residents]*** that I observed during all these years. For example, one of them [a resident] due to his/her cognitive impairment started to pee right in the middle of the common hall, so the other residents started to attack saying *«you are disgusting, you can't do it here, now you're wet and you're standing close to me!»*. What happens? These ***residents [with cognitive impairment]*** don't understand these comments, but they understand that somebody is shouting at them. …and the feelings; they continue having feelings, so it's like they are getting smaller and smaller without clearly understanding what is going on. However, they of course understand non‐verbal language and think like *«they have punished me, they have scolded me, I have done something wrong»*. And all these comments create feelings of discomfort, anxiety, insomnia, and restlessness. *—#15, Nurse*

## 6 Discussion

This qualitative study aimed at understanding the emotions associated with UI in NH residents based on the observations of frontline HCPs. We determined residents' negative feelings towards UI in three main domains: having UI as a long‐lasting condition, experiencing urinary loss and fear of future urinary losses. Furthermore, we identified factors that contributed to alleviating or worsening these negative emotions.

According to the interviewed HCPs, feelings of frustration, shame, anger, impotence, and guilt may be related to the long duration of UI which makes the problem difficult to cope with. Although most residents value having independent bladder function, many of them believe UI is unavoidable and untreatable (Bascur‐Castillo et al. 2019). This belief makes them feel impotent, similar to the feelings reported in our study. The residents hold the view that their condition is a normal and inevitable aspect of ageing, yet they attempt to maintain continence and manage their symptoms. Some adopt self‐management strategies; however, considerable barriers hinder their ability to maintain continence and seek timely treatment (Bascur‐Castillo et al. 2019). Low levels of knowledge about UI appear to be related to a lack of urine continence (Karakaya et al. 2019).

As for experiencing urinary loss, it has been reported that every form of incontinence has psychological consequences: shame, self‐blame, and continuous mental involvement with the condition which could potentially lead to social restrictions (Javanmardifard et al. 2022). A systematic integrative review has shown that self‐stigma is a common perception that older people have towards UI (Yan et al. 2022). They report that feelings of shame, embarrassment, humiliation, and disgust emerge with UI symptoms, and this often leads to miss opportunities of getting support from family members, friends and health professionals (Yan et al. 2022). In the present study, we identified UI‐related negative feelings regarding fear of future episodes of urinary leakage. Those feelings could be related to disappointing people from their social circle who trust them. Concordant with this finding, a qualitative study identified fear was a constant companion of women with UI as the participants were afraid of others' judgements and attitudes towards the condition. The affected women thought that their family and friends would not be willing to have relationships with them due to their incontinence (Javanmardifard et al. 2022). Furthermore, in NHs where HCPs are at the frontline to tackle this issue, the residents feel embarrassed, frustrated and even angry when they have to be cleaned up and changed by them. A qualitative study described a similar phenomenon attributing stigma to the exacerbation of incontinence problems (Murphy et al. 2021). The perceived lack of compassion or understanding from others could increase a sense of threat if an ‘accident’ happens and lead to risk aversion. It seems the culture of secrecy and profound sense of shame represents a barrier for people with incontinence to talk about their condition and seek treatment. Aligned with our findings, a meta‐ethnography study identified that most people with incontinence feel stigmatised and guilty; they commonly feel embarrassed, blame themselves and are worried about the smell (Toye and Barker 2020).

Aside from determining the emotions related to UI, we identified the factors that could alleviate those negative feelings in NH residents. We found that normalising and de‐emphasising the importance of UI makes light of the actual problem. In a similar fashion, another qualitative study with NH staff showed that humour helps minimise some residents' embarrassment, but it does not work for all residents (Ostaszkiewicz et al. 2018). For instance, HCPs may have a close relationship with some residents, with whom joking about UI could be appropriate, whereas other residents may require a different approach because each person is unique (Ostaszkiewicz et al. 2018). Nevertheless, de‐emphasising the importance of UI could be a blessing and a curse because some HCPs have the misconception that incontinence is an unresolvable problem and part of the normal ageing process (Hunter and Wagg 2018; Yan et al. 2023). All in all, increasing awareness on types and treatment for UI is crucial for all HCPs providing direct care for NH residents (Ostaszkiewicz et al. 2018).

Further evidence suggests that some older men cope with UI by reframing it as part of their ‘new normal’, a strategy that appears to lessen its perceived impact on their daily lives. According to Shaw et al. (2024), participants reported that adopting a more accepting mindset—expressed through thoughts such as ‘I've learned to just take UI in stride’ or ‘UI is not the worst thing in the world, but slightly annoying’ – helped them manage the emotional burden associated with the unpredictability of UI (Shaw et al. 2024). These narratives illustrate how cognitive reframing and normalisation of symptoms can function as adaptive coping mechanisms.

The HCPs of our study revealed the bond of trust between them and the residents alleviates the negative emotions derived from UI. Accordingly, a recent qualitative study showed that the confidence between women with UI and HCPs helped them to ‘get a lot off their chest’ (Fu et al. 2023). Therefore, individually planned care is considered a key element in the management of UI. Furthermore, our participants mentioned that little adaptations to time and space of activities in NHs help the residents gain confidence to control their UI. This is consistent with previous research from our group which reported that difficulties in accessing the toilet in NHs caused an increase in the frequency of UI, especially urgency incontinence (Yildirim et al. 2023).

Notwithstanding, we identified two emotional UI‐related worsening factors in NH residents: conflicts among residents and cognitive impairment. Indeed, the impression of others could trigger a variety of negative emotions. Likewise, carers reported in a qualitative study that many people living at home with dementia and incontinence feel acute fear regarding the response of others and cut themselves off from friendship groups to avoid distressing themselves or others (Murphy et al. 2021). Additionally, stigma has been shown to have a double impact when both dementia and incontinence are present in community‐dwelling older individuals (Cole and Drennan 2019).

Yet little is known about how cognitively impaired residents perceive their condition. Some of them may respond with acute anxiety when carers attempt to provide continence care (Ostaszkiewicz 2018). However, despite people with incontinence often expressing that talking to others can be difficult, they claim it could be helpful to tackle UI (Toye and Barker 2020).

### 6.1 Strength and Limitations of the Work

The main limitation of this study is that the residents' emotions were not directly assessed but instead gathered by proxy NH staff because of the pandemic restrictions. Consequently, data collection may have been sensitive to information bias, as the experiences described reflect HCPs' interpretations rather than residents' first‐person accounts. However, conducting interviews with NH residents is challenging even under non‐pandemic conditions due to the high prevalence of cognitive impairment in this frail segment of the population, as demonstrated by a study conducted in the same region (Farrés‐Godayol et al. 2022). In these situations, relying on proxy respondents such as staff or relatives represents an accepted and necessary approach for accessing residents' lived experiences when direct participation is not possible (Kunicki et al. 2023). Importantly, within a Husserlian descriptive phenomenological framework, HCPs can be considered privileged observers of residents' embodied emotional expressions, behavioural cues, and affective reactions that occur during intimate continence‐care interactions. Their close and continuous engagement with residents allows them to witness emotional responses as they naturally unfold in daily life, making their accounts a meaningful and phenomenologically coherent source of insight, particularly when cognitive impairment limits residents' ability to articulate their own experiences.

### 6.2 Recommendations for Future Research

Future research should include different views on the problem, that is, not only HCPs but also stakeholders, relatives and residents, whenever possible. This may contribute to a deep analysis of UI in NHs so that preventive and management interventions in NH settings can be developed and applied.

## 7 Conclusions

The HCPs working in NHs perceive diverse UI‐related negative emotions in residents: feeling impotent, guilty, frustrated, embarrassed, angry, uncomfortable, worried, and insecure. Factors that alleviate those negative feelings are de‐emphasising the importance of UI, time, space adaptations in NHs, and the bond of trust between healthcare professionals and residents. Factors that worsen negative emotions are conflict among residents and cognitive impairment. The importance of this study lies in understanding how UI affects the residents' emotions, suggesting strategies to palliate this problem from a person‐centred approach.

## Author Contributions

J.J.‐R., E.M.‐M., M.Y. and L.C.‐P. designed the study. P.M.‐M. and S.R.‐F. assisted in participant recruitment. Data collection was primarily conducted by M.Y., with J.J.‐R., L.C.‐P. and S.R.‐F. providing support. P.M.‐M. performed the transcriptions, and M.Y. analysed the data. Preliminary findings were presented to all authors for finalising the thematic scheme. V.R.O. and E.M.‐M. wrote the introduction, M.Y. wrote the methodology and results, V.R.O. and J.J.‐R. wrote the discussion and conclusions. S.R.‐F. managed the references and citations. All authors reviewed and approved the final version of the manuscript. S.H. did methodological and linguistic editing.

## Funding

This work was supported by the Hestia Chair from *Universitat Internacional de Catalunya* (grant number BI‐CHAISS‐2019/003), and *Col·legi de Fisioterapeutes de Catalunya* (Catalan Board of Physiotherapists) (grant number R03/19). Javier Jerez‐Roig holds a grant from the Research Council of Lithuania and the Ministry of Education, Science and Sport of the Republic of Lithuania (Project No. S‐A‐UEI‐23‐2).

## Ethics Statement

This study received approval from the Ethics and Research Committee of the University of Vic—Central University of Catalonia (UVic‐UCC) (92/2019 and 109/2020), as well as the Clinical Research Ethics Committee of the Osona Foundation for Health Research and Education (FORES) (2020118/PR249).

## Conflicts of Interest

The authors declare no conflicts of interest.

## Supporting information


**Table S1:** Illustrative coding table showing the development of themes from meaning units.

## Data Availability

The datasets used and/or analysed during this study are available from the corresponding author on reasonable request.
