# Supplementary material for: Emotions Associated With Urinary Incontinence in Nursing Home Residents: A Phenomenological Analysis of Healthcare Professionals' Perspectives
Source: Nurs Open. 2026 Jun 22;13(6):e70620. doi: 10.1002/nop2.70620 (PMC13287329; doi:10.1002/nop2.70620)
Supplement: Supplementary file 1 — Table S1: Illustrative coding table showing the development of themes from meaning units. [file NOP2-13-e70620-s001.docx]

**Supplementary Table S1. Illustrative coding table showing the development of themes from meaning units**

| **Meaning Unit (verbatim quotation)** | **Condensed Meaning Units** | | **Theme** |
| --- | --- | --- | --- |
|  | **Description close to the text** | **Interpretation of the underlying meaning** |  |
| “What an embarrassment that I have wet myself, now you need to clean me…” (#05) | Resident feels embarrassed after urinary loss and needing help | Embarrassment due to loss of control and dependency | Feeling embarrassed |
| “There are people [residents] that accept it in general, but anyways there are moments of frustration when they realise that they couldn’t hold their pee. (#05) | Residents experience frustration when they fail to remain continent | Frustration due to inability to control urine | Feeling frustrated |
| “If they have a urine leakage before arriving at the toilet, they get angry [with themselves].” (#12) | Residents become angry after losing control | Anger directed at oneself after leakage | Feeling angry |
| “They feel very impotent…” (#02); “Especially among the ones [residents] that are cognitively okay, the sensation of impotence affects them seriously.” (#15) | Residents feel powerless due to lack of control | Sense of impotence linked to loss of autonomy | Feeling impotent |
| “I am sorry that you need to change my clothes/diaper…” (#15) | Residents apologise and feel guilty for needing help | Guilt associated with dependency | Feeling guilty |
| “I know they feel uncomfortable… [They are like] *what if I can’t hold my pee?*” (#04) | Residents feel discomfort when anticipating leakage | Discomfort linked to uncertainty and risk | Feeling uncomfortable |
| “[They are like] … *let’s see if I can hold my pee, let’s see if I will get myself wet or not...”* (#07) | Residents worry about possible urinary loss | Worry related to unpredictability of UI | Feeling worried |
| “…sometimes they feel a little bit insecure because they must go running [to the toilet] so the others [the other residents] don’t see that he/she has lost urine.” (#17) | Residents feel insecure in social situations due to UI | Insecurity linked to social exposure | Feeling insecure |
| “Giving the message that it’s not a problem for us [HCPs]… Speaking to them calmly and friendly like it [IU] is not that important, it’s something related to aging, we all will have it at the end.” (#05) | Normalising UI helps residents feel less distressed | Normalisation reduces emotional burden | Alleviating factor: De-emphasising UI |
| “…planning the group activities in spaces that are closer to the toilet … help them [the residents] to feel calm and secure.” (#02) | Environmental adaptations increase comfort and security | Contextual adaptations reduce anxiety | Alleviating factor: Adaptations in activities |
| “The bond of trust… is a great facilitator. Sharing intimate problems [to a person that you trust] is easier [to explain]” (#07) | Trust enables residents to express emotions more easily | Trust facilitates emotional relief | Alleviating factor: Bond of trust |
| “One resident starts saying ‘Uf! Here it smells very bad!’ and the one with UI feels embarrassed.” (#06) | Negative comments from others increase embarrassment | Social conflict exacerbates emotional distress | Worsening factor: Conflict among residents |
| “They [residents with cognitive impairment] don’t understand the [conflicting] comments [of other residents] but they feel they have done something wrong.” (#15) | Cognitive impairment increases emotional vulnerability | Impaired understanding intensifies distress | Worsening factor: Cognitive impairment |
